# Technofunctional Properties and Rheological Behavior of Quinoa, Kiwicha, Wheat Flours and Their Mixtures

**DOI:** 10.3390/molecules29061374

**Published:** 2024-03-20

**Authors:** Nicodemo C. Jamanca-Gonzales, Robert W. Ocrospoma-Dueñas, Yolanda M. Eguilas-Caushi, Rossy A. Padilla-Fabian, Reynaldo J. Silva-Paz

**Affiliations:** Escuela de Ingeniería en Industrias Alimentarias, Departamento de Ingeniería, Universidad Nacional de Barranca, Barranca 15169, Peru; njamanca@unab.edu.pe (N.C.J.-G.); rocrospomad@unab.edu.pe (R.W.O.-D.); yeguilasc@unab.edu.pe (Y.M.E.-C.); rpadillaf@unab.edu.pe (R.A.P.-F.)

**Keywords:** flour mixtures, dough, quinoa, kiwicha, rheology

## Abstract

Wheat flour is a common raw material in the food industry; however, Andean grains, such as quinoa and kiwicha, are gaining popularity due to their quality proteins, fiber, and bioactive compounds. A trend has been observed toward the enrichment of products with these Andean flours, with them even being used to develop gluten-free foods. However, evaluating interactions between raw materials during industrial processes can be complicated due to the diversity of inputs. This study focused on evaluating the technofunctional and rheological properties of wheat, quinoa and kiwicha flours using a simple lattice mixture design. Seven treatments were obtained, including pure flours and ternary mixtures. Analyses of particle size distribution, water absorption index, subjective water absorption capacity, soluble material index, swelling power, apparent density and physicochemical properties were performed. Additionally, color analysis, photomicrographs and Raman spectroscopy were carried out. The results indicate significant differences in properties such as particle size, water absorption and rheological properties between the flours and their mixtures. Variations in color and microstructure were observed, while Raman spectroscopy provided information on molecular composition. These findings contribute to the understanding of the behavior of Andean flours in baking and pastry making, facilitating their application in innovative food products.

## 1. Introduction

Wheat flour is a raw material widely used in the food industry for the production of different baking and pastry products, consisting mainly of complex carbohydrates that provide nutrients. However, Andean grains such as quinoa and kiwicha are becoming highly valued due to the quality of their proteins, fiber content, and the presence of minerals such as calcium and iron [1,2,3]. In addition, they constitute an important source of bioactive compounds, such as phenolic acids and flavonoids [1,4]. Currently, there is a tendency to use their mixtures for enrichment purposes in various innovative products, from quinoa [5] to bread enriched with kiwicha flour [6] or even in the development of foods for celiacs [7]. During industrial processes, it is pertinent to evaluate the interaction effects that raw materials and inputs can have on the final product [8], but these evaluations are often complicated by the incorporation of a varied number of inputs. There are research reports that have evaluated the effect of flour interactions, partially replacing the percentage of wheat flour with different sources of Andean grain flours, such as quinoa and kiwicha [1,2]. An elementary aspect in the processing of all types of baking products is the preparation of the dough, consisting of the mixture of any type of flour and water, the product of which forms a complex system that is both unstable and subject to continuous modification of its physical characteristics through the actions of physical, chemical and biological forces [9], which is studied by rheometry—the amount of deformation that a material experiences when forces are applied.

The microstructure of raw materials such as flour is an elementary aspect in the physical characterization of foods, as it allows us to understand the conformation, morphology and size of the organic components that are part of the food system and to understand their surface structure [10], which is closely linked to their textural properties. Scanning electron microscopy is an immensely popular imaging instrument today [11]. The rheological behavior of foods is considered the study of the deformation and flow of unprocessed raw materials, intermediate or semi-finished products, and final products in order to evaluate the quality preferred by the consumer through correlations among measurements. Rheological and sensory tests, which consist of evaluating the texture of food through optical procedures and the sensations of ingestion and touch, elucidate the structure or composition, as well as the structural changes that occur during the conditioning and processing—both the quantity and the state of the components that constitute it [12]. Furthermore, the rheological properties of foods also serve as a means to control or monitor a process [13].

There are studies aimed at evaluating the rheological behavior of wheat flours and their mixtures with substituted flours, e.g., sweet potato and wheat for making bread [14]; wheat and corn flours [9]; coroba *(Jessenia polycarpa Karst)* [15]; wheat, barley and potatoes in the production of bread [16]; extruded rice flour and barley bagasse [17]; and the interaction effects of quinoa, kiwicha and tarwi flours on pasta properties (peak viscosity, minimum viscosity, retrogradation viscosity and final viscosity) and texture (firmness, consistency, cohesiveness, viscosity index and consistency) of the gels formed in an aqueous system [8]. The incorporation of elements such as salt influences the rheological characteristics of the masses [18]. However, the technofunctional parameters of Andean grains or the interaction between them have not yet been reported. Therefore, considering the interest in studying the behavior of Andean grain flours during the production of baking products, the objective of this work was to evaluate the technofunctional and rheological properties of wheat, quinoa and kiwicha flours using simplex lattice mixture design. This work aims to understand the behavior of these flours and their mixtures in the production of baked products, filling a gap in research on Andean grains.

## 2. Results and Discussion

### 2.1. Technofunctional Properties

Table 1 shows the results of the technofunctional properties of the flours and their different mixtures. A significant difference was found in the particle size index (PTI) (*p* < 0.05) of the different flours, showing variability in their physicochemical properties, which directly influences the derived products [19]. Quinoa flour (T2) has a higher particle size index, followed by wheat flour (T1) and the finest kiwicha flour (T3), while the mixtures present very varied size indexes. These differences are related to the efficiency of grinding in industrial processes; that is, the greater the grinding, the smaller the size achieved, but a wider distribution thereof, i.e., a greater variability within the different particles that make up the fine flour powder [20]. The water absorption index (WAI) presented significant differences (*p* < 0.05), with an inversely proportional relationship with the ITP, T6 being the sample with the lowest WAI value, while T3 constituted the most granulated sample with the highest particle size. These granulometry results are lower than those obtained by Dussán-Sarria et al. [21] in kiwicha flour samples.

The subjective water absorption capacity (SWAC) of the samples presents significant differences (*p* < 0.05) whose results are superior to the IAA with the exception of T7. These values are lower than what was reported in similar samples of kiwicha flour obtained by Dussán-Sarria et al. [21], whose values were influenced by the solid and elastic behavior generated by an interaction between the fiber and starch with the proteins [22] of the flour, mainly gluten in the case of wheat. The soluble material index (SMI) presents a significant difference between the treatments (*p* < 0.05), presenting two well-differentiated groups, the first group consisting of T2, T3, T4, T6 and T7, while T1 and T5 present similarity. In relation to the swelling power (SP), there is a significant difference between the samples (*p* < 0.05), with differences existing, whose values are close to those reported by Dussán-Sarria et al. [21] in kiwicha flours (T3), characterized by its greater prevalence of fibers and starch. The apparent density of the flours constitutes a very important physical property that allows it to be characterized, which, in the case of powdery products, is linked to the porosity of the samples, there being a relationship with the ITP, with a significant difference (*p* < 0.05) between the samples. The apparent density values are very close to those reported by Cerezal Mezquita et al. [23].

### 2.2. Proximate Compositions

Table 2 presents the results of the proximate compositions of the flours with their different mixtures. The proximate composition data show that there is a significant difference in moisture (*p* < 0.05). T1 presented the highest moisture, while T3 showed the lowest content. These values are below what is established by INACAL [24] and INACAL [25], which establish a maximum of 12%. The moisture content in flour is essential for its conservation and is directly related to other components that add up to the total solids in flours. Regarding fat content, our results align with INACAL guidelines [24,25] and closely resemble findings from comparable studies on Andean grains, such as quinoa and cañihua [26]. Notably, significant differences (*p* < 0.05) exist among treatments, with wheat flour exhibiting the lowest fat content and kiwicha flour (T3) registering the highest fat percentage. Importantly, the fat content of T2 falls within the range established by Verena et al. [27]. In terms of crude fiber content, our values are below those recommended for dietary fiber by Reyes García et al. [28]. It is crucial to note that these values may underestimate the true fiber content in foods [29]. Both T2 and T3 show elevated content, while T1, due to being shelled, exhibits lower content, with a significant difference observed among the samples (*p* < 0.05). Protein content falls within the range established by Reyes García et al. [28]. Quinoa and kiwicha, as pseudocereals, are noteworthy for their reserve proteins (60–70% of fractions), such as globulins and albumins, rich in essential amino acids and sulfur [30]. The protein content of T2 falls within the range set by Verena et al. [27], with a significant difference among samples (*p* < 0.05). Total carbohydrates, representing a function of water, fat, ash and protein content, exhibit significant differences among samples (*p* < 0.05). Carbohydrates fall within the expected range of 48.5–82.2%, constituting the main components of pseudocereals in the form of starch, monosaccharides and disaccharides, including glucose, fructose, arabinose, xylose, sucrose and maltose [30].

### 2.3. Physicochemical Parameters and Rheology

In Table 3, the results of the physicochemical, rheological parameters and the Raman spectrum are presented. The pH of the flours and their corresponding mixtures show that there is a significant difference in pH (*p* < 0.05), with T3 having the highest hydrogen ion potential and T2 showing the lowest content. In general, the pH values of flours and their mixtures are located within the group of low-acidity or non-acidic foods (pH of 5.0–6.8) [6]. The acidity of the flours shows significant differences between them (*p* < 0.05). The flour mixtures with different levels of substitutions are below the maximum value of 0.18% in the case of brown flours, while the quinoa and kiwicha flours have a level of acidity higher than the maximum allowed by the Peruvian Technical Standard [31] due to the presence of other components, mainly soluble and insoluble fibers and other organic components, these acidity values of kiwicha flours being close to those obtained by Pascual Chagman and Zapata Huamán [6].

Rheology is important in many productive aspects that cover the physical characteristics of foods; it is linked to the study of how liquid, solid or semi-solid materials deform or flow when a force or tension is applied to them, its evaluation being of vital interest in the production of baking products. The rheological behavior of the flours with their different mixtures indicates that the apparent viscosity (K) of T3 was higher, evidenced by the greater gelatinization of the starch and greater resistance to flow. This was followed by T1, characterized by the presence of globulins and gliadins that form the gluten during the kneading of the flours. Samples T2 and T6 presented the lowest values. The flow index (n) indicated that there are significant differences. Samples T2 and T6 showed less pseudoplastic behavior, and samples T3 and T5 presented more pseudoplastic behavior, suggesting an interaction between wheat, quinoa and kiwicha flours. The incorporation of different percentages of quinoa and kiwicha flours produced changes in the rheology of the dough [22]. Raman spectroscopy is a technique that allows the structural identification of molecules and is being used in the characterization of various materials in their different forms, including thin films, powders, liquids, volumetric samples and even gases, which only require a small amount of sample. Raman spectroscopy is a non-destructive technique [32], thus having significant potential for the characterization of powdery foods such as flour. The samples presented significant differences.

### 2.4. Chromatic Characteristics of Flours with Their Different Mixtures

**Figure 1:**
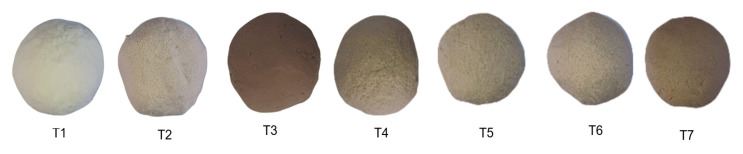
Chromatic characteristics of flour doughs and their mixtures: T1: 100% wheat flour (HT); T2: 100% quinoa flour (HQ); T3: 100% kiwicha flour (HK); T4: 33.33% HT, 33.33% HQ, 33.33% HK; T5: 66.67% HT, 16.67% HQ, 16.67% HK; T6: 16.67% HT, 66.67% HQ, 16.67% HK; and T7: 16.67% HT, 16.67% HQ, 66.67% HK.

Table 4 presents the chromatic values of the flours and doughs made from the mixtures studied. In relation to the color of the flours and their mixtures, with their respective masses, the L* (whiteness) value of wheat flour was higher than that of kiwicha and quinoa flours. The average values of the color parameters evaluated indicate that wheat, quinoa, kiwicha flours and their mixtures are different in color. The color is influenced by the presence of pigments linked to phytochemicals such as total phenolic compounds and total flavonoids [33]. There is a significant difference between the color results; however, the results for quinoa are close to those obtained by Dussán-Sarria et al. [6]. The formation of the dough, obtained by mixing flour and water, causes the color parameters to be modified, decreasing the L value, while the parameters a, b and C increase. In Figure 1, the different color shades of the masses with their respective mixtures are observed. The color of quinoa flour (T2) is similar to that reported by Arteaga et al. [34], being slightly different due to the ecotype used in the study.

**Figure 2:**
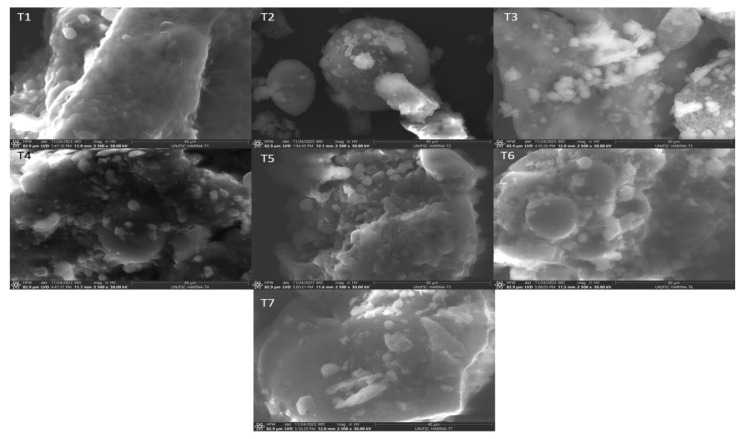
Scanning electron microscopy images at 40 µm of flours and their mixtures: T1: 100% wheat flour (HT); T2: 100% quinoa flour (HQ); T3: 100% kiwicha flour (HK); T4: 33.33% HT, 33.33% HQ, 33.33% HK; T5: 66.67% HT, 16.67% HQ, 16.67% HK; T6: 16.67% HT, 66.67% HQ, 16.67% HK; and T7: 16.67% HT, 16.67% HQ, 66.67% HK.

Flours are multicomponent systems [35], e.g., carbohydrates, proteins, fats and dietary fiber [28], so structurally, they are complex matrixes made up of starch particles of various sizes together with smaller fragments that usually correspond to portions of proteins. In Figure 2, the images of the flours are observed using electron microscopy. The flours are structured based on starch granules immersed in a protein matrix. Thus, the microphotographs of the flours show the significant presence of starches, mainly in T1, presenting spherical shapes, while in the other samples, it is observed in a lower proportion. Flours present starches within a carbohydrate matrix cover—the major component of flours [36]. Wheat starch granules have spherical shapes, with a bimodal size distribution with large lenticular granules, ~25 µm, and small spherical ones of <10 µm in diameter [37]. The starches of quinoa flour show that the granules have a polygonal shape, with a granule size of approximately 1.22 µm, which coincides with the range of 1–2 µm, as reported by Bernal Bustos et al. [38].

### 2.5. Microphotographs of the Flours

Figure 2 shows the microstructure of the different flours analyzed by scanning electron microscopy (SEM). Wheat flour (T1) exhibits starch granules and a protein matrix that form a voluminous three-dimensional network of protein chains. On the other hand, quinoa flour (T2) has starch granules with shapes that vary from polygonal and angular to irregular. As for kiwicha flour (T3), a cellular structure is observed that allows for the identification of cell walls, starch, proteins, and fibers with variations in shape and size. The dimensions of the starch granules are different for each type of flour; wheat granules have a range between 0.2 and 30 μm, while those of quinoa and kiwicha are mainly in the range of 0.4 to 20 μm, as reported previously [39] for quinoa flour. Furthermore, quinoa starch aggregates have spherical or oblong shapes with sizes between 10 and 30 μm [40]. In contrast, samples T4, T5, T6 and T7 show less visible starch granules in the microstructure, making their discernment difficult due to the presence of multiple components in the system. On the other hand, proteins play a crucial role in baking quality, with glutenins and gliadins being responsible for the formation of gluten that confers viscoelasticity during kneading [35]. During this process, proteins interact through hydrogen, ionic, hydrophobic and covalent bonds to form a cross-linked network [41].

### 2.6. Evaluation of Flours with Raman Spectroscopy

Raman analysis is a vibration-based spectroscopy technique that identifies the components of multicomponent samples without the need to separate the elements individually [42,43]; it is suitable for the qualitative analysis of solid products [44,45,46]. However, it can be affected by interferences such as background fluorescence, which affects detection, in addition to the analysis problem and difficult quantitative analyses due to non-linear bias [47]. The Raman spectra are observed from the flours with their mixtures. Differences are evident between the treatments, with sample T6 showing greater intensity, reaching peaks around 47,100 u.a, followed very closely by T5; samples T7 and T2 were located far below, T4 and T3 were intermediate, while T1 was the lowest, with a peak of 4500 u.a. The trends of the spectra are similar, with the exception of T7 and T3, which are samples based on kiwicha flour, whose behavior is likely due to natural pigments. When the samples are analyzed as fine powder, there is a lot of interference from the fluorescence background, with a direct influence on the vibrational and rotational energy levels of the chemical components of the flours, mainly starch. Ramirez et al. [48] reported similar values for jicama starch (*Pachyrhizus erosus*), with peaks of highly varied intensity between shifts of 400 to 1500 cm^−1^, reaching a maximum intensity of 1200 u.a.

### 2.7. Correlation between the Properties of Flours and Doughs

**Figure 3:**
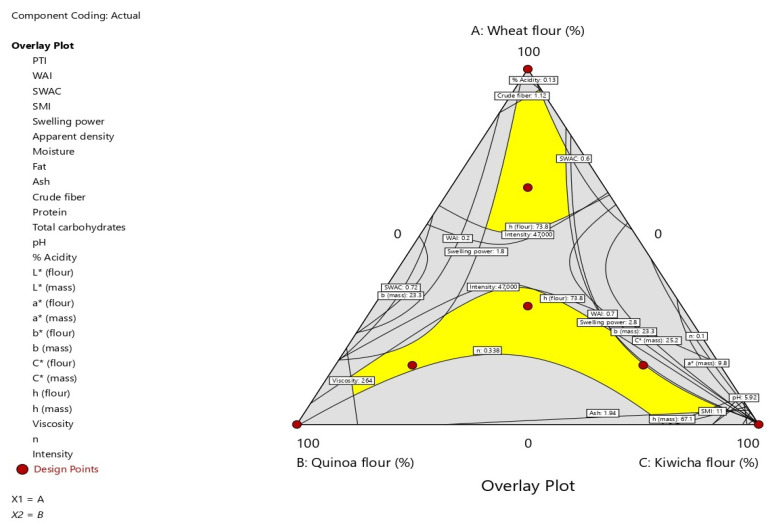
Overlay graph of the effects of wheat, quinoa and kiwicha flour mixtures on the properties of the flours and their masses.

The analysis found that the mixture design on the properties of water absorption rate, subjective water absorption capacity, swelling power, apparent density, protein, total carbohydrates, flux index and Raman intensity do not correlate significantly with the properties of the flours and their mixtures studied. However, the particle size index, soluble material index, moisture, fat, ash, crude fiber, pH, acidity, apparent viscosity, color parameters of flour (L*, a*, b*, C*) and mass (L *, a*, b*, h*) were significant (*p* < 0.05). Figure 3 shows the overlay plots of the significant response variable effects, where the yellow regions represent the best responses [49].

The location of the variables on the graph represents their direct influence on the closest component. The percent acidity is directly influenced by the increase in wheat flour in the mixture, while the apparent viscosity is influenced by the greater proportion of kiwicha flour in the mixture. A higher percentage of kiwicha increases the pH, SMI and color of the product. That is, depending on the flours, their characteristics vary independently; in the same way, when making the mixtures, these parameters that predominate independently are reduced. Based on the significant response variables, linear and quadratic predictive mathematical models were constructed, which are shown in Table 5. All significant variables were represented by a linear model (first order), except for b* (doughs), which was fitted to a quadratic model. From these equations, the behavior can be obtained when increasing or reducing the flours studied so that it can be analyzed which variables to prioritize in a food product. Various studies have shown that the combination of wheat, quinoa and kiwicha flours can improve properties such as yeast performance, mechanical properties of the dough, water absorption in the dough, shelf life, structure and texture of the baked product [50,51,52].

## 3. Materials and Methods

### 3.1. Obtaining Flours and Their Different Mixtures

Commercial wheat flour (HT) special panettone brand, bulk quinoa (HQ) and bulk kiwicha (HK) were used, purchased in the central market of Barranca, Peru, and processed by industrial grinding, conventionally using hammer mills. The milling process in wheat consists of the separation of the germ endosperm and pericarp, followed by a gradual reduction in particle size [53], while the production of quinoa and kiwicha flours uses whole grains [8], including the following stages: reception and inspection of the material, magnetic separation of impurities, cleaning, conditioning (adjustment of moisture content), grinding and screening, sieving and bagging.

For the generation of mixtures, the simplex lattice mixture design was used, obtaining 7 treatments, consisting of 3 pure components (T1: 100.00% HT, T2: 100.00% HQ and T3: 100.00% HK) and 4 ternary mixtures (T4: 33.33% HT, 33.33% HQ, 33.33% HK; T5: 66.67% HT, 16.67% HQ, 16.67% HK; T6: 16.67% HT, 66.67% HQ, 16.67% HK; T7: 16.67% HT, 16.67% HQ, 66.67% HK).

### 3.2. Technofunctional Properties

#### 3.2.1. Particle Size Distribution or Granulometry (PSD)

PSD was determined by sieving 100 g samples of each flour in a sifter (WS Tyler Rotap, model RX-29-16, series 12528, Columbus, OH, USA) with a set of 9 Tyler series sieves of numbers 16 (1.18 mm), 20 (0.850 mm), 30 (0.600 mm), 40 (0.425 mm), 50 (0.300 mm), 60 (0.250 mm), 100 (0.150 mm), 200 (0.075 mm) and 270 (0.053 mm) through constant vibrational movement. With 30 min of sieving time, each fraction of the meshes was separated, and the fractions retained in the different meshes were weighed according to AOAC 965.22 [54]. The distribution was reported as % retention in each mesh based on the initial flour sample.

#### 3.2.2. Particle Size Index (PTI)

The particle size index is an indicator of flour quality; high values indicate finer flours that are related to more cohesive doughs. The ITP was calculated according to the method modified by Platt [55]. Each factor used depends on the U.S. sieve series number (0.2, No. 20 mesh; 0.4, No. 40 mesh; 0.6, No. 60 mesh; 0.8, No. 80 mesh; 1.0, No. 100 mesh; 2.0, No. 200 mesh; 2.7, No. 270 mesh; and bottom) and the % of retention of each mesh obtained according to the particle size distribution analysis.

#### 3.2.3. Water Absorption Index (WAI)

For WAI determination, we used the method modified by Gonzalez Vera [56]. The modification consisted of the use of distilled water at room temperature (25 °C) and reduction of the sample quantity. First, 0.5 g of sample was added to a 15 mL centrifuge tube, then 7.5 mL of distilled water at room temperature was added. The suspension was shaken for 30 min, followed by centrifugation at 5000 rpm for 30 min. The supernatant was placed on a previously tared glass plate and evaporated in an oven at 105 °C (Binder brand, Tuttligen, Germany). The weight of the gel was recorded as well as the precipitate. The IAA was expressed as g of water/g of dry sample.

#### 3.2.4. Subjective Water Absorption Capacity (SWAC)

The subjective water absorption capacity is the amount of water absorbed by flour to obtain a dough of appropriate consistency for dough preparation and is a subjective test. The methodology described by Platt [55] was used. First, 100 g of flour was weighed, adding water gradually, kneading gently by hand until a dough of good consistency was obtained. The amount of water added was recorded as the water absorption capacity of the flour in ml of water/100 g of flour.

#### 3.2.5. Soluble Material Index (SMI)

SMI was determined using the method modified by Gonzalez Vera [56], which was calculated based on the weight of the soluble material present in the supernatant (used to determine the IAA) and the amount of initial sample.

#### 3.2.6. Swelling Power (SP)

The swelling power was determined according to the methodology proposed by G. Sandoval et al. [16], using 0.5 g of sample of each flour, estimated based on the values of the weight of the gel and weight of the dry supernatant.

### 3.3. Proximate Compositions

#### 3.3.1. Moisture

Moisture was determined by the gravimetry method or weight loss due to drying, established by INACAL [57]. A clean, completely dry crucible was weighed with 5 g of sample, then placed in the oven (Binder brand, Goettingen, Germany) at 130 ± 3 °C for 1 h. It was then placed in a desiccator, allowed to cool and weighed on an analytical balance (Sartorious brand, Goettingen, Germany).

#### 3.3.2. Fat

Fat determination was carried out in a Solvent Extractor (Velp Scientifica, model SER148, Usmate Velate, Italy), using petroleum diethyl ether as the fat extraction solvent at 40–60 °C with a maximum residual evaporation of 10 mg/L.

#### 3.3.3. Ash 

Ash was determined using a muffle furnace (Therm Concept brand, model KL15/11, Bremen, Germany) by incineration and calcination at 550 °C for 3–4 h [28].

#### 3.3.4. Protein

The protein was determined from the total nitrogen value determined by the Kjelhdal method, multiplied by a factor of 6.25 [28], according to the AOAC method, method 97.09, using a Velp Kjeldahl Analyzer consisting of a JP Recirculating water aspirator, DKL Heating digester and Smi-Automatic Distillation Unit UDK 139 (Velp Scientifica, model 139, Usmate Velate, Italy).

#### 3.3.5. Total Carbohydrates

The total carbohydrate values were calculated by the difference in dietary fiber value. It is obtained by subtracting the weight in g of the macrocomponents from 100, according to the following formula: Total carbohydrates (g) = 100 − (protein + fat + moisture + ash) [28].

#### 3.3.6. Crude Fiber

Crude fiber was determined by acid–alkaline hydrolysis. It is based on sequential treatment with acids and alkalis under standardized conditions, although the dietary fiber content is significantly undervalued, using the gravimetric method [29].

### 3.4. Physicochemical Parameters

#### 3.4.1. Apparent Density (AD)

AD was determined according to the method proposed by Gonzalez Vera [56], placing it in a funnel on a universal support with a cast iron container at the bottom with an internal diameter of 29.84 mm, a height of 49.70 mm and a capacity of 32 mL. Once the flour was carefully poured into the funnel, the passage gate was opened, and the flour was allowed to flow freely. With the help of a ruler, the excess flour was scraped off, and the container of known volume was weighed. To obtain the apparent density of the flour, the weight of the flour was divided by the volume of the container, expressed in g/mL.

#### 3.4.2. Ph

The pH of the sample was determined using the AOAC 943.02 method [58]; 25 ± 1 g was weighed in 100 mL of distilled water, which was added to a beaker. The supernatant was carefully decanted, and the pH was immediately measured with the previously calibrated potentiometer or pH meter.

#### 3.4.3. Acidity

Total acidity was determined by gravimetry, according to the AACC 02-31.01 method cited in INACAL [59], for which 10 g of sample was weighed and placed in a 300 mL Erlenmeyer, adding 100 mL of distilled water. The contained suspension was shaken every 10 min for 1 h, then filtered until a minimum filtrate volume of 50 mL was obtained. Then, 50 mL of filtrate was placed in a 125 mL Erlenmeyer flask, and 1 mL of phenolphthalein solution was added. It was titrated with 0.1 N NaOH solution until the color change to redcurrant occurs, which should persist for 30 s, using Titration equipment (Thermo Fisher Scientific Inc. model Titronic brand 500, Madrid, Spain). The acidity was expressed in % referred to sulfuric acid and calculated based on 15% humidity.

### 3.5. Colorimetric Parameters and Image Analysis

#### 3.5.1. Color

The instrumental color was determined using a colorimeter (PCE Instruments brand, model CSM 3, Albacete, Spain) with an observation angle of 10° and Illuminant D65 at 420 nm. The values of the coordinates L*, a* and b* were measured, the equipment was calibrated with a white reflecting dish or plate (L* = 97.58, a* = 0.16 and b* = 1.71), and the parameters determined were L* = 0 [black] and L* = 100 [white]; a*(−a* = green and + a = red), b*(−b = blue and +b = yellow). Here, 3 g per sample was used, and 10 repetitions were carried out for each of the flours. The Chroma (C*) and tone (h*) values were determined. The following equations were used, as proposed by Dussán-Sarria et al. [21]. The color of the dough was determined from the dough obtained with the subjective water absorption capacity test (items 2.2.3). The simulated color was determined using the Color Selector application at the following link: https://es.rakko.tools/tools/30/ (accessed on 20 December 2023).

#### 3.5.2. Photomicrographs

The images were recorded with a scanning electron microscope (Thermo scientific brand, model Prisma E, Praha, Czech Republic) incorporated with a quasor II model X-ray generator. The photomicrographs were recorded at 2500 × 30 kV. The weight of the sample was 0.1000 ± 0.0001 g.

### 3.6. Analysis with Raman Spectrometry

Raman Spectrometry was conducted according to the method proposed by Zamora-Peredo et al. [60] using a spectrometer consisting of an Avantes Sens Line Spectrometer (Avantes, model ULS.TEC, Apeldoorn, The Netherlands), Power Supply (Avantes, model laser, Apeldoorn, The Netherlands) and Invisible Laser Radiation at 785 nm and 499 mW (Avantes, model laser, Apeldoorn, The Netherlands), whose data were processed with Ava Soft 8.14 Software Full. Intrinsic silicon was used as the calibration standard. The reading of the samples was carried out with a time of 1 s at a power of 1000 mW.

### 3.7. Rheological Flow Properties of Flour Dough

The rheological properties were evaluated in triplicate using a compact modular rheometer (Anton Paar brand, model MCR 72, Graz, Austria) and the linear flow curve test type. The run parameters were 25 points, constant duration, point interval 5 s, run time 125 s, with a variable cut rate in a linear ramp and a final frequency of 50 s^−1^. The masses used in the analysis were those prepared with the results of the subjective water absorption capacity methodology, with modifications proposed by Belén C et al. [15]. Data management was performed using RheoCompass TH software, version 1.31.70-Release.

### 3.8. Statistical Analysis

For the data obtained from the technofunctional and rheological properties, the mixture design was applied, and the analysis of variance (ANOVA) was applied to determine the influence of the mixtures and which mathematical model (linear, quadratic, special cubic and full cubic) was significant (*p* < 0.05) and fit the results. In addition, a comparison of multiple means (Tukey HSD) was performed. All statistical treatments were carried out with the R Project 4.3.3 and Rstudio version 2023.12.1 software using the “agricolae” and “mixexp” packages. For the graphics and the best fit of the experimental results to the mathematical models, the Stat-Ease 360^®^ Trial version software (Stat-Ease Inc., Minneapolis, MN, USA) was used.

## 4. Conclusions

The results provide valuable information about the behavior of the flours studied. The diversity in particle size, water absorption capacity and rheological properties highlight the complexity of the interactions between these ingredients during industrial processes. Kiwicha flour improves the technofunctional and rheological properties, although it generates variations in the color of the dough. In the proximal composition, the quinoa flour and the wheat mixture with 16.67%, quinoa 66.67% and kiwicha 16.67% presented higher values compared to the other samples. The evaluation of physicochemical properties, color and microstructure offers a complete vision of the characteristics of flours and their mixtures. The correlation analysis revealed significant associations among the various properties, such as particle size index, soluble material index, humidity, fat, pH, acidity, apparent viscosity and color parameters through the linear and quadratic mathematical model, which allows us to predict this behavior, contributing to a deeper understanding of the complex interaction of variables in flour mixtures. A product with higher crude fiber, acidity and SWAC content should have a higher percentage of wheat flour. For greater viscosity, it should have a greater proportion of quinoa flour and lower kiwicha content with wheat. Higher values of color, ash, pH and SMI should have a higher content of kiwicha and lower quinoa with wheat. This study contributes to knowledge about the potential of Andean grain flours in the food industry, supporting its application in the development of innovative and enriched food products, whether independently or in mixtures.

## Figures and Tables

**Table 1:** Technofunctional properties of flours with their different mixtures.

Sample	Particle Size Index (PTI)	Water Absorption Index (g Water/g Dry Sample)	Subjective Water Absorption Capacity (mL Water/100 g Flour)	Soluble Material Index (SMI)	Swelling Power (SP)	Apparent Density (g/mL)
T1	209.62 ± 0.20 ^b^	0.35 ± 0.04 ^cd^	0.61 ± 0.01 ^e^	6.82 ± 0.51 ^b^	1.90 ± 0.07 ^cd^	0.84 ± 0.01 ^c^
T2	211.78 ± 0.31 ^a^	0.58 ± 0.02 ^abc^	0.69 ± 0.01 ^b^	9.83 ± 0.03 ^a^	2.49 ± 0.03 ^ab^	0.86 ± 0.01 ^bc^
T3	122.76 ± 0.01 ^g^	0.69 ± 0.02 ^a^	0.72 ± 0.01 ^a^	10.63 ± 0.14 ^a^	2.78 ± 0.03 ^a^	0.88 ± 0.01 ^ab^
T4	195.08 ± 0.01 ^c^	0.54 ± 0.03 ^abc^	0.66 ± 0.01 ^cd^	10.05 ± 0.33 ^a^	2.42 ± 0.07 ^abc^	0.88 ± 0.01 ^a^
T5	193.93 ± 0.04 ^d^	0.44 ± 0.01 ^bcd^	0.64 ± 0.01 ^d^	8.04 ± 0.08 ^b^	2.13 ± 0.04 ^bcd^	0.87 ± 0.01 ^ab^
T6	192.16 ± 0.01 ^e^	0.29 ± 0.14 ^d^	0.67 ± 0.01 ^bc^	9.63 ± 0.69 ^a^	1.85 ± 0.33 ^d^	0.88 ± 0.01 ^a^
T7	137.71 ± 0.01 ^f^	0.66 ± 0.06 ^ab^	0.64 ± 0.01 ^d^	10.95 ± 0.08 ^a^	2.72 ± 0.14 ^a^	0.88 ± 0.01 ^a^
Coefficients						
HT	208.07	0.35	0.60	6.79	1.89	0.84
HQ	210.23	0.57	0.68	9.81	2.48	0.86
HK	121.21	0.69	0.72	10.60	2.77	0.88
HT × HQ	94.79	−1.63	0.34	−5.41	−3.83	0.13
HT × HK	−24.49	2.15	−0.22	5.59	4.93	0.02
HQ × HK	−58.69	−1.04	−0.34	6.67	−2.01	0.09
Model						
Linear	0.01	0.16	0.08	0.01	0.11	0.28
Quadratic	0.96	0.41	0.36	0.32	0.43	0.09

Values expressed in mean ± standard deviation. Values with different lowercase letters are significantly different according to the Tukey test (*p* < 0.05). Wheat flour (HT), quinoa flour (HQ) and kiwicha flour (HK).

**Table 2:** Proximate compositions of the flours with their different mixtures.

Sample	Moisture (g)	Fat (g)	Ash (g)	Crude Fiber (g)	Protein (g)	Total Carbohydrates
T1	13.59 ± 0.23 ^a^	2.28 ± 0.09 ^d^	0.35 ± 0.10 ^c^	1.12 ± 0.17 ^b^	10.41 ± 0.03 ^a^	72.17 ± 0.44 ^ab^
T2	10.54 ± 0.26 ^c^	5.81 ± 0.55 ^ab^	1.94 ± 0.07 ^a^	2.09 ± 0.67 ^ab^	10.67 ± 0.08 ^a^	68.96 ± 0.13 ^d^
T3	7.57 ± 0.19 ^e^	7.77 ± 1.03 ^a^	1.90 ± 0.39 ^a^	2.93 ± 0.14 ^a^	9.77 ± 0.05 ^c^	70.07 ± 0.65 ^cd^
T4	10.77 ± 0.02 ^c^	5.28 ± 0.13 ^bc^	1.53 ± 0.14 ^ab^	1.88 ± 0.58 ^ab^	9.28 ± 0.04 ^d^	71.26 ± 0.51 ^bc^
T5	11.86 ± 0.22 ^b^	3.78 ± 0.01 ^cd^	1.03 ± 0.04 ^bc^	1.42 ± 0.17 ^b^	8.43 ± 0.08 ^e^	73.48 ± 0.26 ^a^
T6	10.69 ± 0.05 ^c^	5.55 ± 0.21 ^bc^	1.54 ± 0.18 ^ab^	1.46 ± 0.19 ^b^	10.07 ± 0.09 ^b^	70.69 ± 0.29 ^bc^
T7	9.22 ± 0.06 ^d^	6.53 ± 0.58 ^ab^	1.85 ± 0.01 ^a^	2.13 ± 0.20 ^ab^	8.51 ± 0.04 ^e^	71.77 ± 0.40 ^b^
Coefficients						
HT	13.58	2.28	0.35	1.10	10.36	72.26
HQ	10.52	5.81	1.94	2.07	10.62	69.05
HK	7.55	7.77	1.90	2.90	9.72	70.15
HT × HQ	−1.24	0.01	−1.09	−1.28	−1.34	5.18
HT × HK	−1.06	0.01	2.77	1.73	−14.62	11.41
HQ × HK	3.26	−0.01	−0.62	−3.60	3.48	−2.71
Model						
Linear	<0.01	<0.01	0.01	0.02	0.57	0.11
Quadratic	0.52	0.91	0.14	0.59	0.37	0.58

Values expressed in mean ± standard deviation. Values with different lowercase letters are significantly different according to the Tukey test (*p* < 0.05). Wheat flour (HT), quinoa flour (HQ) and kiwicha flour (HK).

**Table 3:** Physicochemical parameters, rheological composition and Raman spectrum of the flours with their different mixtures.

Sample	pH	% Acidity (Expressed in Sulfuric Acid)	Apparent Viscosity (K) (Pa·s)	Flow Index (n)	Intensity to 200 cm^−1^
T1	5.64 ± 0.03 ^c^	0.13 ± 0.01 ^e^	1244.90 ± 21.10 ^b^	0.26 ± 0.01 ^b^	3555.50 ± 4.95 ^f^
T2	5.29 ± 0.01 ^e^	0.51 ± 0.01 ^a^	264.17 ± 0.18 ^d^	0.33 ± 0.01 ^a^	42,336.00 ± 131.00 ^c^
T3	5.91 ± 0.01 ^a^	0.24 ± 0.01 ^cd^	1980.60 ± 41.80 ^a^	0.10 ± 0.01 ^c^	29,391.00 ± 151.00 ^e^
T4	5.66 ± 0.04 ^c^	0.27 ± 0.02 ^c^	998.60 ± 76.90 ^c^	0.26 ± 0.01 ^b^	34,501.00 ± 183.00 ^d^
T5	5.73 ± 0.04 ^bc^	0.19 ± 0.01 ^d^	810.80 ± 75.90 ^c^	0.16 ± 0.02 ^c^	46,111.00 ± 533.00 ^a^
T6	5.50 ± 0.04 ^d^	0.36 ± 0.01 ^b^	266.70 ± 16.60 ^d^	0.32 ± 0.02 ^a^	46,989.00 ± 16.60 ^a^
T7	5.81 ± 0.01 ^ab^	0.27 ± 0.01 ^c^	938.40 ± 46.70 ^c^	0.24 ± 0.03 ^b^	44,163.00 ± 26.90 ^b^
Coefficients					
HT	5.64	0.13	1211.46	0.26	4792.32
HQ	5.29	0.51	230.73	0.33	43,572.82
HK	5.92	0.24	1947.16	0.10	30,627.82
HT × HQ	0.45	−0.31	410.74	−0.69	0.01
HT × HK	0.36	0.21	−1827.44	−0.30	0.01
HQ × HK	−0.21	−0.12	−2472.25	1.16	−75,607.77
Model					
Linear	0.01	0.01	0.03	0.07	0.31
Quadratic	0.48	0.13	0.73	0.12	0.61

Values expressed in mean ± standard deviation. Values with different lowercase letters are significantly different according to the Tukey test (*p* < 0.05). Wheat flour (HT), quinoa flour (HQ) and kiwicha flour (HK).

**Table 4:** Chromatic properties of flours with their different mixtures.

Treatment	Flour Color Parameters					
	L*	a*	b*	C*	h*	Color Simulation
T1	93.33 ± 1.03 ^a^	0.68 ± 0.28 ^f^	9.50 ± 0.82 ^e^	9.53 ± 0.83 ^e^	85.97 ± 1.28 ^a^	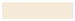
T2	88.23 ± 0.20 ^b^	2.62 ± 0.12 ^e^	12.59 ± 0.12 ^d^	12.87 ± 0.15 ^d^	78.27 ± 0.44 ^b^	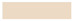
T3	79.72 ± 1.42 ^d^	5.16 ± 0.37 ^a^	17.79 ± 0.46 ^a^	18.53 ± 0.54 ^a^	73.85 ± 0.73 ^d^	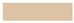
T4	86.56 ± 0.26 ^b^	3.64 ± 0.13 ^c^	14.02 ± 0.06 ^c^	14.49 ± 0.08 ^c^	75.45 ± 0.42 ^cd^	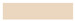
T5	86.68 ± 1.04 ^b^	3.51 ± 0.23 ^cd^	12.86 ± 0.21 ^d^	13.32 ± 0.25 ^d^	74.75 ± 0.75 ^cd^	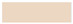
T6	88.24 ± 0.49 ^b^	3.02 ± 0.16 ^de^	12.13 ± 0.13 ^d^	12.50 ± 0.13 ^d^	76.04 ± 0.74 ^c^	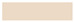
T7	83.63 ± 0.43 ^c^	4.39 ± 0.08 ^b^	15.51 ± 0.07 ^b^	16.12 ± 0.09 ^b^	74.21 ± 0.19 ^cd^	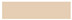
Coefficients						
HT	93.28	0.70	9.47	9.50	85.84	
HQ	88.18	2.64	12.57	12.84	78.14	
HK	79.67	5.18	17.77	18.51	73.72	
HT × HQ	−16.30	7.94	4.29	5.57	−38.41	
HT × HK	−20.63	9.12	13.62	14.98	−33.91	
HQ × HK	28.77	−8.36	−13.65	−14.88	27.75	
Model						
Linear	0.01	0.03	0.01	0.01	0.18	
Quadratic	0.22	0.18	0.28	0.22	0.28	
	**Color parameters of doughs**					
T1	85.54 ± 0.58 ^a^	2.02 ± 0.18 ^f^	16.07 ± 0.20 ^e^	16.19 ± 0.22 ^f^	82.84 ± 0.54 ^a^	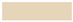
T2	77.77 ± 0.52 ^b^	6.32 ± 0.13 ^e^	20.51 ± 0.25 ^d^	21.46 ± 0.26 ^e^	72.87 ± 0.30 ^b^	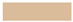
T3	54.67 ± 0.78 ^f^	9.76 ± 0.12 ^a^	23.23 ± 0.51 ^a^	25.19 ± 0.45 ^a^	67.20 ± 0.59 ^f^	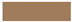
T4	67.49 ± 0.67 ^d^	7.40 ± 0.12 ^c^	22.58 ± 0.36 ^ab^	23.76 ± 0.38 ^bc^	71.86 ± 0.03 ^cd^	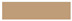
T5	72.66 ± 0.49 ^c^	6.99 ± 0.18 ^d^	21.99 ± 0.19 ^bc^	23.08 ± 0.23 ^cd^	72.36 ± 0.32 ^bc^	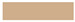
T6	72.98 ± 0.46 ^c^	7.23 ± 0.14 ^cd^	21.32 ± 0.18 ^cd^	22.51 ± 0.21 ^d^	71.26 ± 0.19 ^d^	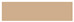
T7	59.45 ± 0.34 ^e^	8.67 ± 0.09 ^b^	22.54 ± 0.19 ^ab^	24.15 ± 0.21 ^b^	68.95 ± 0.09 ^e^	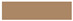
Coefficients						
HT	85.51	2.07	16.07	16.21	82.69	
HQ	77.73	6.37	20.52	21.48	72.73	
HK	54.63	9.81	23.23	25.21	67.05	
HT × HQ	−17.45	15.23	20.64	23.47	−30.61	
HT × HK	−41.17	11.91	19.06	20.75	−24.29	
HQ × HK	9.35	−11.07	−15.69	−17.68	22.33	
Model						
Linear	0.01	0.03	0.14	0.09	0.03	
Quadratic	0.09	0.29	0.01	0.06	0.40	

Values expressed in mean ± standard deviation. Values with different lowercase letters are significantly different according to the Tukey test (*p* < 0.05). Wheat flour (HT), quinoa flour (HQ) and kiwicha flour (HK).

**Table 5:** Predictive mathematical models that indicate the effect of each component of the mixture and their interactions on the color parameters, properties of the flours and properties of the gels formed when mixing wheat flour (HT), quinoa flour (HQ) and kiwicha flour (HK).

Parameters	Predictive Mathematical Equation	R^2^
Particle Size Index	ITP = 210.75 × HT + 211.77 × HQ + 118.78	0.9219
Soluble Material Index	IMS = 7.00 × HT + 10.05 × HQ + 11.21 × HK	0.9904
Moisture	M = 13.53 × HT + 10.62 × HQ + 7.65 × HK	0.9952
Fat	F = 2.28 × HT + 5.80 × HQ + 7.77 × HK	0.9999
Ash	A = 0.44 × HT + 1.91 × HQ + 2.00 × HK	0.9668
Crude fiber	CF = 1.02 × HT + 1.81 × HQ + 2.75 × HK	0.8653
pH	pH = 5.69 × HT + 5.31 × HQ + 5.94 × HK	0.9689
Acidity	A = 0.12 × HT + 0.49 × HQ + 0.23 × HK	0.9839
Apparent viscosity	k = 1050.04 × HT + 47.82 × HQ + 1689.64 × HK	0.8133
L* (Flour)	L* = 91.81 × HT + 88.36 × HQ + 79.70 × HK	0.9105
a* (Flour)	a* = 1.53 × HT + 2.88 × HQ + 5.46 × HK	0.8190
b* (Flour)	b* = 10.19 × HT + 12.38 × HQ + 17.89 × HK	0.9397
C* (Flour)	C* = 10.35 × HT + 12.70 × HQ + 18.68 × HK	0.9359
L* (Doughs)	L* = 82.11 × HT + 76.01 × HQ + 52.12 × HK	0.9365
a* (Doughs)	a* = 1.53 × HT + 2.88 × HQ + 5.46 × HK	0.8190
b*_m_ (Doughs)	b*_m_ = 16.07 × HT + 20.52 × HQ + 23.23 × HK + 20.64 × HT × HQ + 19.06 × HT × HK-15.69 × HQ × HK	0.9999
h* (Doughs)	h* = 79.91 × HT + 71.49 × HQ + 66.03 × HK	0.8160

The significant variables (*p* < 0.05) were selected for the construction of the predictive mathematical model of the quadratic and linear types.

## Data Availability

The data are contained in the article.
